# Molecular Structure and Variation Characteristics of the Plastomes from Six *Malus baccata* (L.) Borkh. Individuals and Comparative Genomic Analysis with Other *Malus* Species

**DOI:** 10.3390/biom13060962

**Published:** 2023-06-08

**Authors:** Xun Wang, Ruifen Zhang, Daru Wang, Chen Yang, Yawen Zhang, Mengyi Sui, Jian Quan, Yi Sun, Chunxiang You, Xiang Shen

**Affiliations:** 1 State Key Laboratory of Crop Biology, National Research Center for Apple Engineering and Technology, College of Horticultural Science and Engineering, Shandong Agricultural University, Taian 271018, China; 2018110226@sdau.edu.cn (X.W.); 2020110302@sdau.edu.cn (D.W.); 2021120417@sdau.edu.cn (C.Y.); 2022110294@sdau.edu.cn (Y.Z.); 2022110283@sdau.edu.cn (M.S.); 2 Qingdao Academy of Agricultural Sciences, Qingdao 266100, China; zhangruifen316@gmail.com; 3 China National Botanical Garden, Beijing 100093, China; quanjian@chnbg.cn (J.Q.); sunyi@chnbg.cn (Y.S.)

**Keywords:** *Malus baccata*, chloroplast genome, sequence similarity, variation and diversity, phylogeographic, comparative genomic analysis

## Abstract

*Malus baccata* (L.) Borkh. is an important wild species of *Malus*. Its rich variation types and population history are not well understood. Chloroplast genome mining plays an active role in germplasm identification and genetic evolution. In this study, by assembly and annotation, six complete cp genome sequences, ranging in size from 160,083 to 160,295 bp, were obtained. The GC content of stable IR regions (42.7%) was significantly higher than that of full length (36.5%) and SC regions (LSC-34.2%, SSC-30.4%). Compared with other *Malus* species, it was found that there were more sites of polymorphisms and hotspots of variation in LSC and SSC regions, with high variation sites including *trnR/UCU*-*atpA*, *trnT/UGU*-*trnL/UAA*, *ndhF*-*rpl32* and *ccsA*-*ndhD*. The intraspecific and interspecific collinearity was good, and no structural rearrangement was observed. A large number of repeating elements and different boundary expansions may be involved in shaping the cp genome size. Up to 77 or 78 coding genes were annotated in the cp genomes of *M. baccata*, and high frequency codons such as UUA (Leu), GCU (Ala) and AGA (Arg) were identified by relative synonymous codon usage analysis. Phylogeographic analysis showed that 12 individuals of *M. baccata* clustered into three different groups with complex structure, whereas variant xiaojinensis (M.H. Cheng & N.G. Jiang) was not closely related to *M. baccata* evolutionarily. The phylogenetic analysis suggested that two main clades of different *M. baccata* in the genus *Malus* were formed and that I and II diverged about 9.7 MYA. In conclusion, through cp genome assembly and comparison, the interspecific relationships and molecular variations of *M. baccata* were further elucidated, and the results of this study provide valuable information for the phylogenetic evolution and germplasm conservation of *M. baccata* and *Malus*.

## 1. Introduction

*Malus baccata* (L.) Borkh., belonging to the genus *Malus*, is considered a large group of wild species [1,2]. It is native to China and has a wide range of natural distributions, including Northeast China, North China and Southwest China [3,4]. *M. baccata* has good fertility and a certain degree of resistance, so it is used in the production of grafting seedling rootstock and apple breeding parents [5]. Due to the differences in ecological environment and the influence of species adaptability, *M. baccata* has accumulated abundant variation types, such as broad leaves, hanging branches, early flowering, large fruit and so on [6,7]. The high genetic diversity gives a better chance of survival and development for *M. baccata* and also allows important data to be gathered for evolutionary relationship analysis and conservation biology research [8].

In addition to *M. baccata*, the genus *Malus* includes a large number of wild and cultivated species, such as *M. ioensis* (Alph. Wood) Britton, *M. florentina* (Zuccagni) C.K. Schneid., *M. yunnanensis* (Franch.) C.K. Schneid., *M. sikkimensis* (Wenz.) Koehne, *M. hupehensis* (Pamp.) Rehder as wild species and *M. spectabilis* (Aiton) Borkh., *M. halliana* Koehne, *M. prunifolia* (Willd.) Borkh., *M. asiatica* Nakai and *M. domestica* (Suckow) Borkh. as cultivated species [7,9,10]. Because of self-incompatibility and extensive interspecific hybridization, species classification and phylogenetic identification of *Malus* are very complicated [11]. Molecular marker sequences based on nuclear and organelle genomes have been used to study population evolution [12,13,14]. The chloroplast genome is maternally inherited and highly conserved, which has obvious advantages in the study of intraspecific variation and interspecific evolution [15]. In 26 cp genome evolutionary trees, *M. prunifolia*, *M. micromalus* and *M. baccata* are more closely related than *M. yunnanensis* [16]. Based on the phylogenetic branches of chloroplast genomes, *M. sieversii* and *M. sylvestris* may be the ancestors of cultivated apple [17].

Considering that there are few studies on the intraspecific variation of *M. baccata* and interspecific relationship of *Malus* species, plastid genome evolution and matrilineal heredity characteristics of six *M. baccata* from different geographical sources were analyzed in this paper. We mainly try to explore the following questions: (1) What are their chloroplast genome characteristics and differences? (2) How are the repeated sequences and boundary junction points distributed in the cp genomes of *M. baccata*? (3) What are the nucleotide polymorphisms and variation sites of the cp genome of *Malus* genus and how do we further classify the structure between different individuals based on the data of the organelle variation in *M. baccata*? (4) What is the evolutionary relationship between *M. baccata* and *Malus* species based on different chloroplast genome sequence sets and other comparative genome analysis?

## 2. Materials and Methods

### 2.1. Sample Extraction and Genome Assembly

Six germplasms of *M. baccata* (S1~S6) from different regions of China (Table S1) were transplanted in Qingdao Apple Rootstock Research and Development Center, Qingdao Academy of Agricultural Sciences and subjected to standard growth and reproduction with careful water and fertilizer management. After leaf collection and DNA extraction (CTAB method), library construction (DNA fragmentation, end repair, A-tail addition, ligation adaptor and PCR enrichment) and DNA sequencing were conducted on the Illumina HiSeq X platform (PE 150 bp). For chloroplast genome assembly, the original reads were assembled with the GetOrganelle v1.7.5 program [18] with the parameters set to ‘-R 15, -k 21, 45, 65, 85, 105,121’. Then, the full-length chloroplast sequence was obtained by a manual check. The GenBank numbers of the chloroplast genomes from the 6 *M. baccata* germplasms were generated (OQ362999~OQ363004) by submitting the sequence to the NCBI database. In addition, the plastid genome data of different species of *Malus* and other genera were also downloaded from the GenBank database for comparative genomic analysis, including *M. baccata* O1~O6 (KX499859, MK896774, MK411001, MK571561, OM232791, OM232793), *M. baccata* var. xiaojinensis X1~X4 (MK434915, OM232782, OM232794, OM232809), *M. sieboldii* (MT593044), *M. prunifolia* (KU851961), *M. mandshurica* (MW115596), *M. honanensis* (MW115594), *M. toringoides* (MT483999), *M. prattii* (MH929090), *M. hupehensis* (MK020147), *M. rockii* (MZ984214), *C. maximowiczii* (*Crataegus*, MZ494512), *P. pyrifolia* (*Pyrus*, KX450877), *P. communis* (*Pyrus*, KX450879), *P.* x *bretschneideri* (*Pyrus*, KX450880), *P. yedoensis* (*Prunus*, KU985054), *P. zippeliana* (*Prunus*, MK168018), *P. mume* (*Prunus*, MN101214) and *P. trichostoma* (*Prunus*, OP598113).

### 2.2. Homology Analysis of Chloroplast Genome

GC content calculations were completed on the CGView/Proksee website (https://proksee.ca/, accessed on 2 January 2023) [19]. The chloroplast genome similarity between *M. baccata* and different *Malus* species was analyzed using Circoletto software v.07.09.16 (http://tools.bat.infspire.org/circoletto/, accessed on 2 January 2023). Geneious was used to characterize collinearity and the rearrangement of chloroplast genomes in *Malus*, and Mauve/progressive Mauve was selected as the alignment algorithm [20].

### 2.3. Annotation and Characteristics of Chloroplast Genome

The lookup of repeated sequences was performed in MISA-web (MIcroSAtellite identification tool, https://webblast.ipk-gatersleben.de/misa/, accessed on 5 January 2023), TRF (tandem repeats finder, https://tandem.bu.edu/trf/home, accessed on 6 January 2023, trf File Match = 2 Mismatch = 7 Delta = 7 PM = 80 PI = 10 Minscore = 50 Maxperiod = 500) and REPuter (https://bibiserv.cebitec.uni-bielefeld.de/reputer, accessed on 7 January 2023) [21,22]. MISA was used for SSR identification, and the parameter was set as mononucleotide = 10, dinucleotide = 5, trinucleotide = 4, tetranucleotide = 3, pentanucleotide = 3, hexanucleotide = 3; REPuter was used for LSR search, and the parameter was set to hamming distance = 3, minimal repeat size = 30.

PGA (plastid genome annotator) was used for regional (LSC, SSC, IRA, IRB) division and gene (CDS, tRNA, rRNA) annotation of chloroplast genomes [23]. The length and boundary of the genes were examined manually. In addition, confusion regarding gene names was corrected. The accuracy of the annotation results was further confirmed in CPGAVAS2 [24] and CPGView (chloroplast genome viewer) [25]. Finally, the correct annotation files (genbank documents) were submitted to the OGDRAW website (https://chlorobox.mpimp-golm.mpg.de/OGDraw.html, accessed on 4 January 2023) to draw organelle genome maps and complete the visualization of genomic characteristics [26].

Codon usage bias–relative synonymous codon usage (RSCU value) and other important indicators that reflect codon adaptability and usage patterns were compared and analyzed in CodonW (http://codonw.sourceforge.net/, accessed on 10 January 2023), with the parameters set to Option_4: Codon usage indices and Option_12: Select all.

### 2.4. Variation Features of Chloroplast Genomes

Nucleotide diversity of the whole chloroplast genome was calculated using the MAFFT strategy (progressive method, FFT-NS-2 algorithm) and DnaSP (DNA polymorphism analysis) [27]. The boundary patterns and junction sites of the four regions (LSC, IRB, SSC, IRA) of chloroplast genomes were demonstrated using the IRscope tool (https://irscope.shinyapps.io/irapp/, accessed on 6 February 2023) [28]. The variation hotspot analysis of the chloroplast genome for *M. baccata* and other *Malus* species was carried out in the mVISTA system (https://genome.lbl.gov/vista/mvista/submit.shtml, accessed on 6 February 2023) [29]. *M. baccata* S1 was designated as the reference genome and the Shuffle-LAGAN program was chosen as the alignment option.

### 2.5. Phylogeographic and Comparative Genomic Analysis

With *M. baccata* O2 (MK896774.1) as the reference genome, 12 sequences of *M. baccata* chloroplast genomes (S1~S6, O1~O6) were compared and the core SNP sites were generated (Snippy, https://github.com/tseemann/snippy, accessed on 20 February 2023). Freebayes (https://github.com/freebayes/freebayes, accessed on 20 February 2023), vcftools, bcftools and SnpEff (http://pcingola.github.io/SnpEff/, accessed on 20 February 2023) were used to merge, extract, filter and annotate VCF files. SNP/INDEL trees (NJ-trees) of chloroplast genomes were constructed and displayed in VCF2Dis (https://github.com/hewm2008/VCF2Dis, accessed on 20 February 2023), fneighbor (http://emboss.toulouse.inra.fr/cgi-bin/emboss, accessed on 20 February 2023) and MEGA X (https://megasoftware.net/, accessed on 20 February 2023). Plink v1.90 (www.cog-genomics.org/plink/1.9/, accessed on 20 February 2023) was the main software for the principal component analysis, and the related result graph was drawn using the R package (ggplot2). The calculation and construction of the haplotype network were analyzed in the software packages DnaSP (http://www.ub.edu/dnasp/, accessed on 15 February 2023) and PopART (https://popart.maths.otago.ac.nz/download/, accessed on 15 February 2023). HomBlocks process (https://github.com/fenghen360/HomBlocks, accessed on 22 February 2023), RAxML-NG (https://github.com/amkozlov/raxml-ng, accessed on 22 February 2023) and Mrbayes (http://nbisweden.github.io/MrBayes/, accessed on 22 February 2023) were downloaded for cp genome alignment and phylogenetic analysis of *M. baccata* and other species [30,31,32]. Among them, RAxML-NG (v1.1) was used for the construction of maximum likelihood tree (parameters: –model GTR + G –bs-trees 1000) and Mrbayes (v3.2.7) was used for Bayesian inference (parameters: ngen = 1,000,000 samplefreq = 1000 nruns = 4 stopval = 0.01). The common coding sequences of the chloroplast genomes of 32 species (24 *Malus* and 8 other species) were compared (MAFFT), pruned (Gblocks) and combined (Concatenation) in the PhyloSuite (v1.2.2) analysis process (http://phylosuite.jushengwu.com/, accessed on 1 March 2023) [33]. Reconstruction of the maximum clade credibility tree and assessment of species divergence time were completed in the BEAST2 program (http://www.beast2.org/, accessed on 3 March 2023) with the parameters set to ‘GTR subst model, Gamma site model, optimised relaxed clock, 50,000,000 chain length and 2 independent runs’ [34]. LogCombiner, Tracer v1.7.2 [35], TreeAnnotator and FigTree v1.4.4 [36] were used for tree merging, ESS (effective sample size) detection (if the ESS is greater than 200, it indicates that the MCMC process could converge and the result is reliable), tree generation and visualization, respectively. The fossil records information and secondary calibration values (log normal prior distribution) used in this analysis are [37]: (1) the divergence estimate age between *Prunus* and Maleae was at 79.7 MYA [38]; (2) the fossil of *Prunus* was dated to ~55 MYA; (3) the date for the divergence of Stem *Crataegus* was ~35 MYA [39,40].

## 3. Results

### 3.1. Chloroplast Genome Composition and Structure of M. baccata (L.)

After DNA sequencing (Illumina paired-end 150 bp) and assembly (the processes of starting from reads), six complete chloroplast genome sequences were obtained. The non-repeating six plastid sequences were assigned registration numbers (OQ362999~OQ363004). The average base coverage produced during assembly was estimated to be between 367.3× and 429.3×, whereas kmer coverage values were about 112.4 to 131.7 (Table S1). The length of their chloroplast genome was between 160,083 bp (S1) and 160,295 bp (S4), which is close to that of the previously reported species (Table 1). Chloroplast DNA is composed of four regions (SC (LSC and SSC) and IRs (IRA and IRB)), which together form a circular molecular structure. By comparing the size of each part of the six sequences, it can be seen that IR regions were basically the same (26,353 or 26,354 bp), whereas the LSC regions of different individuals had greater changes than the SSC regions (Table 1). Overall, the stability and variability of sequence length play a role in shaping chloroplast genome size.

The GC content and GC skew reflect the DNA density and stability of the genome. Through calculation and comparison of the *M. baccata* chloroplast genomes, it can be found the GC content of the whole genome or each section was basically identical for the six sequences provided in this experiment and the other six sequences previously published in the NCBI (Table 1 and Figure S1). In the 12 samples, the GC content in the IR regions (42.7%) was significantly higher than that in the LSC (34.2%), and the lowest content was in the SSC (30.4%). In addition, Figure S1 also shows that they have similar GC skew. The above results indicate that chloroplast partitioning stability is very important for chloroplast structure.

### 3.2. Homology and Similarity of Chloroplast Genomes in M. baccata and Other Malus Species

**Figure 1:**
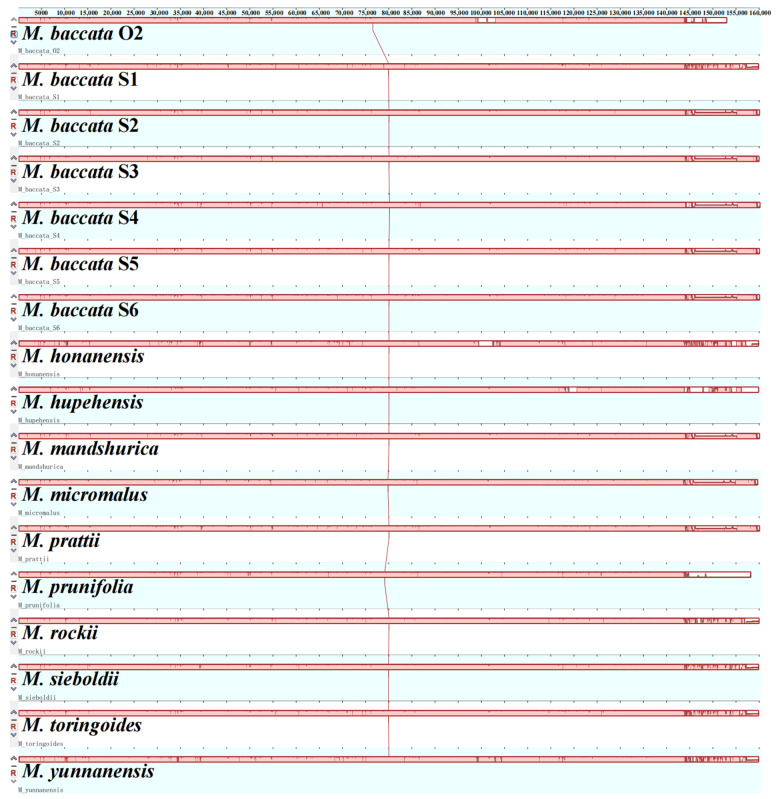
Collinearity analysis of the cp genome of *M. baccata* and different *Malus* species. Comparison of the homology of the six assembled chloroplast genomes (S1~S6) of *M. baccata* were analyzed using O2 as reference.

The homology of species can be better understood by global genome comparison. With O2 as the reference genome, the six chloroplast genomes sequenced in this study showed high homology, which was not only reflected in the reverse repetition region but also in other regions. As shown in Figure 1, a large number of similar fragments are scattered throughout the chloroplast genome. At the same time, a comparison of 7 *M. baccata* and 10 other species of *Malus* showed that chloroplast genomes had good collinearity and no structural rearrangement events were detected (Figure 1). Furthermore, the same phenomenon was found by comparing the chloroplast genomes of different species of *Malus* (Figure S2). What is different is that the interspecific similarity is different in different species. For example, when both are compared with *M. baccata* S1, the similarity effect of *M. toringoides* is better than that of *M. kansuensis*.

### 3.3. Annotation of Chloroplast Genomes in M. baccata

#### 3.3.1. Identification of Repeat Sequences

**Figure 2:**
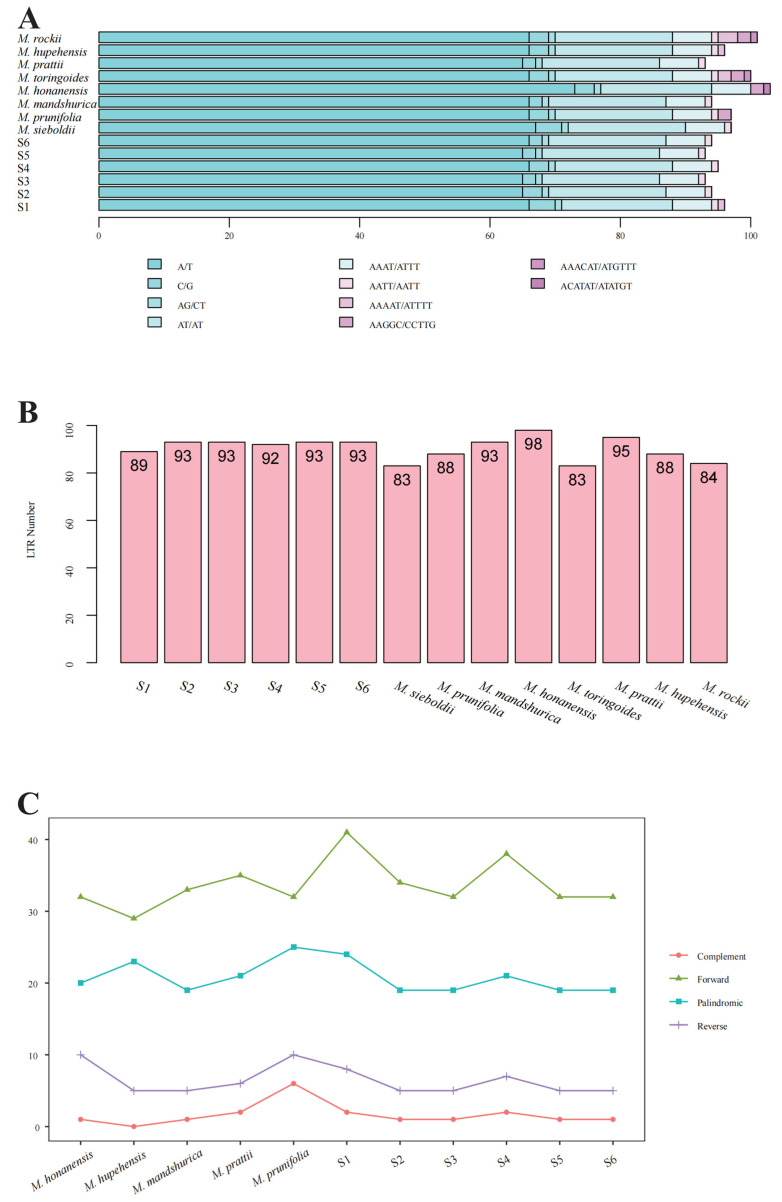
The number and composition of repeats in the cp genome of *M. baccata* and other species. (**A**) Proportion and distribution of SSRs (mono-, di-, tri-, tetra-, penta- and hexanucleotide repeats); (**B**) Long tandem repeats in 14 *Malus* cp genomes; (**C**) Comparison of four types (C, F, P and R) of LSRs in different chloroplast genomes.

The repeats in the chloroplast genome mainly include the following categories: SSRs (simple sequence repeats), LTRs (long tandem repeats) and LSRs (large sequence repeats, dispersed repeats). A large number of repeating elements were identified in the chloroplast genomes of six *M. baccata* species (Table 2) and other different species of *Malus* based on their identifying characteristics. In these cp genomes, *M. honanensis* has the largest number of SSRs (103), followed by *M. rockii* (101), *M. toringoides* (100) and so on (Figure 2A). The number of SSRs from *M. baccata* S1 to S6 was between 93 (*M. baccata* species S3 and S5) and 96 (*M. baccata* species S1). The ‘ACATAT/ATATGT’ SSR type was only identified in the *M. honanensis* cp genome. *M. prunifolia*, *M. honanensis*, *M. toringoides* and *M. rockii* had two ‘AAGGC/CCTTG’ components. Among the 14 cp genomes, the number of LTRs was concentrated between 83 (*M. sieboldii*, *M. toringoides*) and 98 (*M. honanensis*) (Figure 2B). Of the six *M. baccata* species, S2, S3, S5 and S6 had 93 LTR repeats, followed by S4 (92) and S1 (89). LSR type of repeat was a repetitive sequence scattered throughout the chloroplast genome and was involved in the stability and size of the chloroplast genome. LSRs were made up of four types: C (Complement), F (Forward), P (Palindromic) and R (Reverse). F was the most abundant LSR element, followed by P and R. The largest cumulative total for the four LSR components (C, F, P and R) was in *M. baccata* S1, which had 75. In addition, *M. baccata* species S3, S5 and S6 and *M. hupehensis* had the same number of LSRs, which was 57 (Figure 2C).

#### 3.3.2. Gene Recognition

**Figure 3:**
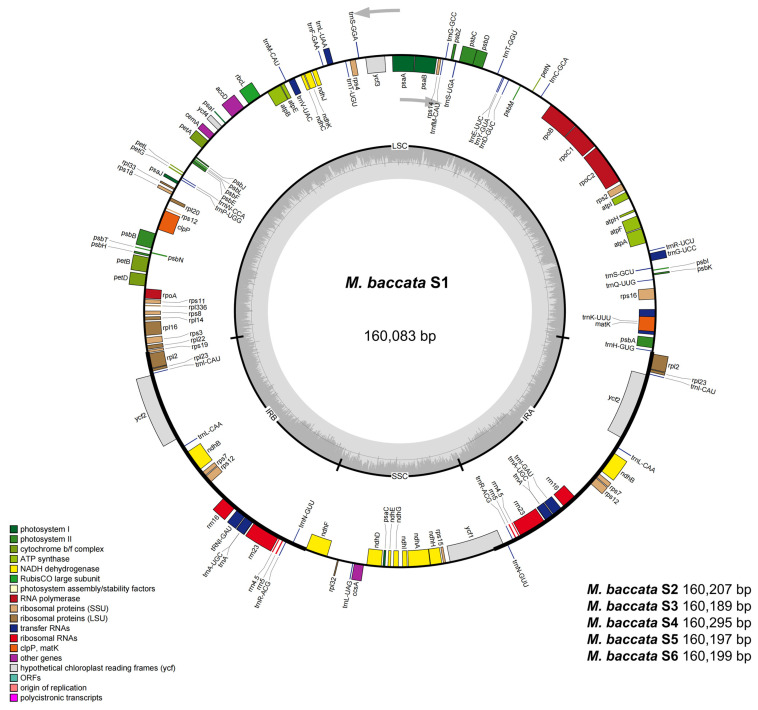
Structure and composition of the cp genome of *M. baccata*. The inner ring represents the four constituent regions of the chloroplast, and the different types of genes are arranged in order in the outer ring. Gray arrows represent transcription directions.

**Figure 4:**
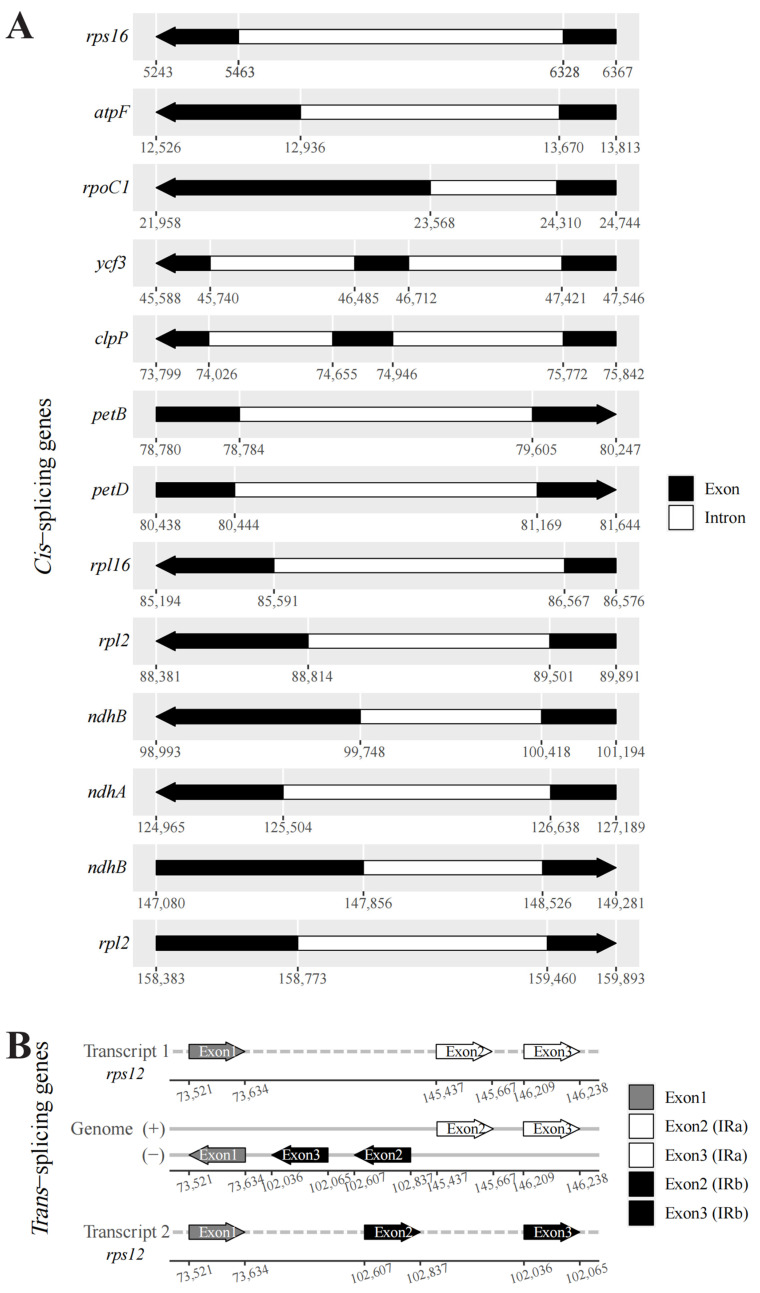
*Cis*-splicing and *trans*-splicing genes in the *M. baccata* (S1) cp genome. (**A**) Genome location and intron composition of cis-splicing genes; (**B**) Schematic map of the trans-splicing gene *rps12* (three unique exons) in the chloroplast genome.

Chloroplast genome annotation is indispensable for the study of gene function, and the objects identified mainly include the CDS (coding sequence), tRNA (transfer RNA) and rRNA (ribosomal RNA). The chloroplast genes of *M. baccata* species S1~S6 were annotated with reference sequences already available. They all had for rRNAs (*rrn4.5*, *rrn5*, *rrn16* and *rrn23*); the type of tRNA was 29 (S1, S3, S6) or 30 (S2, S4, S5), and the number of annotated CDS was 77 (S2, S5, S6) or 78 (S1, S3, S4) (Table 2). Of all the genes, most were single copy (72 CDS, 23 tRNA); however, there were six CDS genes (*ycf2*, *rps7*, *rps12*, *rpl23*, *rpl2* and *ndhB*), seven tRNA genes (*trnV-GAC*, *trnR-ACG*, *trnN-GUU*, *trnL-CAA*, *trnI-GAU*, *trnI-CAU* and *trnA-UGC*) and for rRNA genes (*rrn23*, *rrn16*, *rrn5* and *rrn4.5*) in two copies. For these coding sequences, most of them were involved in photosynthesis, self-replication and essential chloroplast functions, such as participating in c-type cytochrome synthesis (*ccsA*), ATP synthesis (*atpA*, *atpB*, *atpE*, *atpF*, *atpH*, *atpI*), making up the photosystem (*psa*, *psb*) and subunits of the ribosome (*rpl*, *rps*) (Figure 3). By summarizing the two types of genes with introns in the coding sequence, it can be found that there are 11 *cis*-splicing genes (*rps16*, *atpF*, *rpoC1*, *ycf3*, *clpP*, *petB*, *petD*, *rpl16*, *rpl2*, *ndhB* and *ndhA*) and 1 *trans*-splicing gene (*rps12*), among which 2 *cis*-splicing genes (*ycf3* and *clpP*) and the *trans*-splicing gene (*rps12*) have two introns (Figure 4, and Table S2).

#### 3.3.3. Codon Usage Pattern of Chloroplast Genes

**Figure 5:**
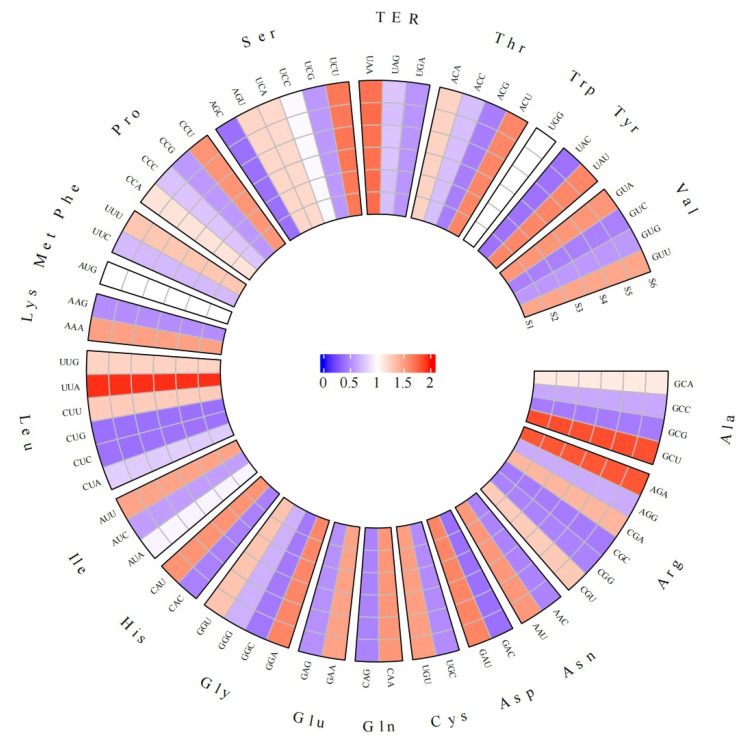
RSCU analysis of cp CDS in *M. baccata*. The outer ring indicates the different codon types, and the scale in the inner ring represents the RSCU value size.

Codon usage bias reflects the differences in gene evolution and expression. Codon usage patterns can be well understood by comparing the ENC (effective number of codons), CAI (codon adaptation index), CBI (codon bias index), FOP (frequency of optimal codons) and RSCU (relative synonymous codon usage). The effective codon numbers of the six chloroplast genomes were about 49, indicating that they had a certain usage bias. In addition, they had similar CAI, CBI and FOP values (Table 3). The RSCU values show that UUA (Leu), GCU (Ala) and AGA (Arg) are used relatively frequently, whereas UAC (Tyr), CUC (Leu), AGC (Ser), CUG (Leu) and GAC (Asp) are used less (Figure 5). In addition, isoleucine favors AUU, serine favors UCU, and the stop codon favors UAA.

### 3.4. Variation and Diversity of Chloroplast Genomes in M. baccata and Other Malus Species

#### 3.4.1. Nucleotide Polymorphism in Whole Chloroplast Genomes

**Figure 6:**
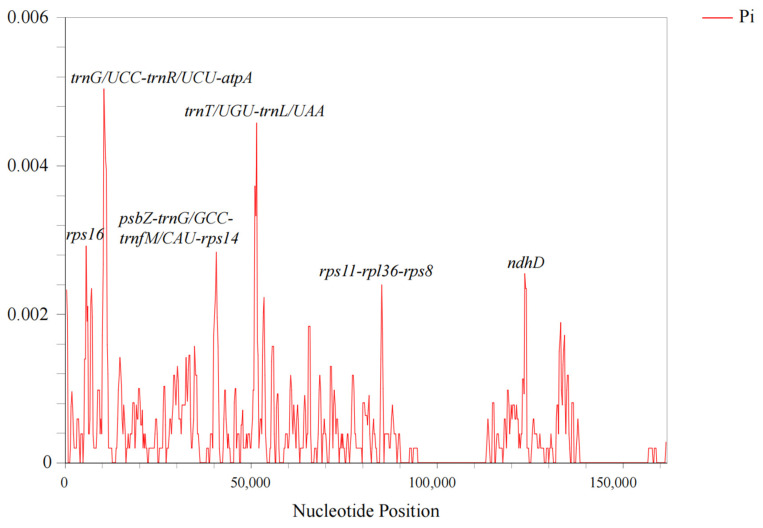
Nucleotide polymorphism map of cp genomes of different *Malus* species. The vertical axis represents the nucleotide polymorphism values.

The examination of nucleotide variation regions in the genome is helpful for species identification and marker development. Using the DnaSP tool, the nucleotide polymorphisms of 17 chloroplasts in the genus *Malus*, including *M. baccata* S1~S6, were calculated. Through sliding window analysis, it can be seen that the polymorphism of the whole genome is varied and that there are distinctions in different regions (Figure 6). The regions with larger differences include *trnG/UCC*-*trnR/UCU*-*atpA*, *trnT/UGU*-*trnL/UAA*, *rps16* (LSC region) and *nhdD* (SSC region), etc.

#### 3.4.2. Chloroplast Genome Boundary Analysis

**Figure 7:**
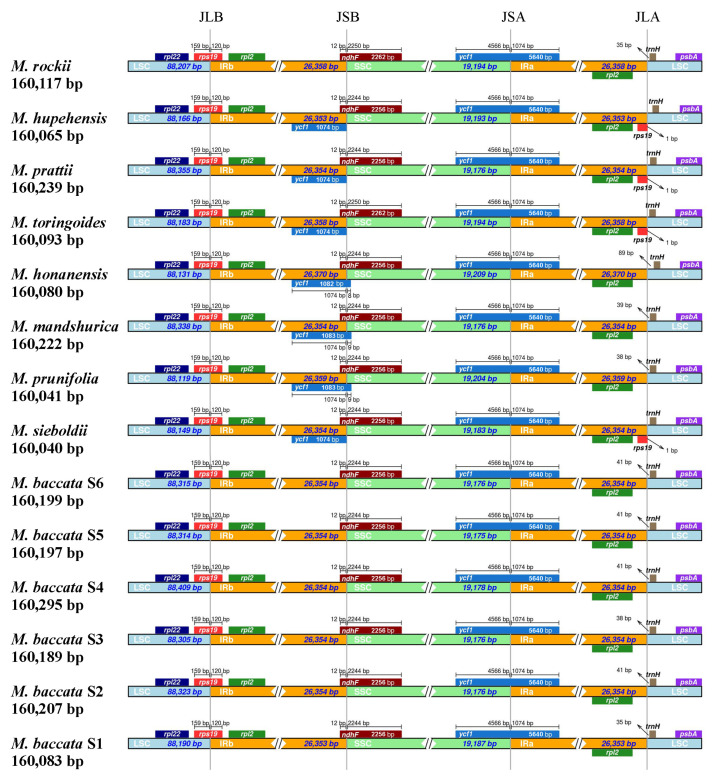
Expansion and contraction of chloroplast boundaries in *Malus*. JLB, JSB, JSA and JLA represent the connecting points of SC and IR regions, i.e., the boundaries.

The extension and contraction of chloroplast genome boundaries reflects genomic evolution and shapes size variation. The conserved IR and the SC formed four connection points, namely JLA (IRa–LSC), JLB (LSC–IRb), JSA (SSC–IRa) and JSB (IRb–SSC). In the chloroplast genomes, the *rps19* gene in the JLB region was stable and consistent (LSC region—159 bp, IRb region—120 bp) (Figure 7). In JSB junction site, there was no *ycf1* pseudogene for *M. baccata* S1~S6 and *M. rockii*, and the offset of *ycf1* to the SSC region is also different for different species (*M. honanensis*—8 bp, *M. mandshurica* and *M. prunifolia*—9 bp). The *rps19* gene in the JLA region was only annotated in *M. hupehensis*, *M. prattii*, *M. toringoides* and *M. sieboldii* (Figure 7). In addition, the distance of *trnH* gene from the IRa side was not completely consistent in different *Malus* species.

#### 3.4.3. Hotspots of Variation in the Chloroplast Genome

The hotspots of variation in the chloroplast genome were analyzed on the mVISTA website. As shown in the Figure S3, in addition to several regions with high polymorphism variation mentioned above, there are also several mutation hotspots. It is worth noting that the variations were slight in the coding regions of the genes and most of the variations were located in the non-coding regions, especially in the intergene regions. Compared with the conservative IR regions, there were more variation hotspots in the SC regions, especially in the LSC region. For LSC, *trnG-UCC*-*trnR-UCU*, *trnR-UCU*-*atpA*, *atpH*-*atpI*, *petN*-*psbM*, *trnT-GGU*-*psbD*, *trnT-UGU*-*trnL-UAA*, *ndhC*-*trnV-UAC* and *petB* could be used as markers of high variation (Figure S3). Additionally, *ndhF*-*rpl32*, *ccsA*-*ndhD* and *ndhA* in the SSC region could be selected as candidate regions for development markers.

### 3.5. Phylogeographic and Comparative Genomic Analysis

#### 3.5.1. Phylogeographic Structure Based SNPs and INDELs

**Figure 8:**
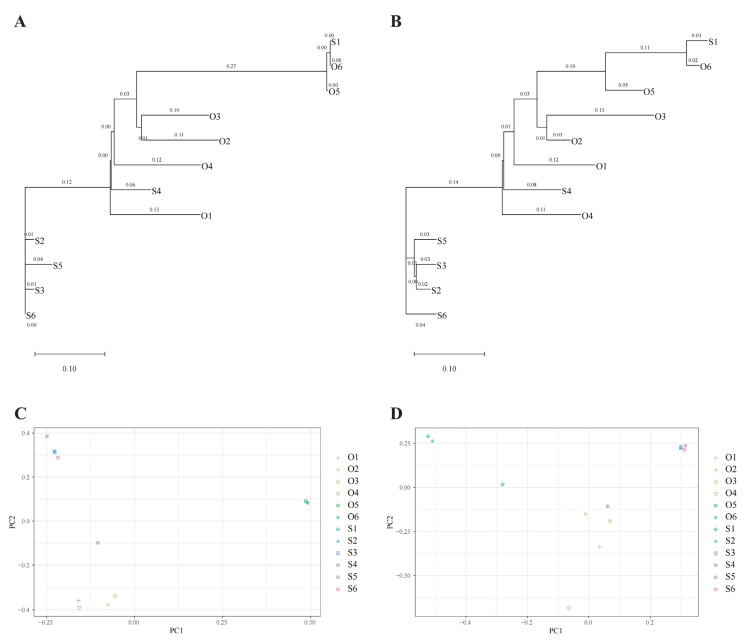
Phylogeographic structure of *M. baccata* based on chloroplast genome variation. (**A**) Topology tree based on population SNP variations; (**B**) Topology tree based on INDELs; (**C**,**D**) Principal component structure diagrams based on SNP and INDEL, respectively.

Based on the reference genome (MK896774), 12 chloroplast genomes of *M. baccata* were tested for variation to explore their relationship composition and lineage structure. A total of 78 SNPs and 109 INDELs were found and extracted for building the evolutionary trees and principal components. As can be seen from Figure 8A, the evolutionary tree based on SNPs is mainly divided into three main branches. The first branch includes S1, O6 and O5, the second branch includes O3, O2, O4, S4 and O1 and the third branch includes S2, S5, S3 and S6. This result is also well reflected in the PCA analysis (Figure 8C). The tree topology structure (Figure 8B) and principal component analysis (Figure 8D) based on INDELs, in general, also shows a consistent and similar phenomenon to the results from the SNP analysis; however, under this clustering condition, the relationship between S1 and O6 was closer than that between S1 and O5 (Figure 8B,D).

#### 3.5.2. Haplotype Analysis

**Figure 9:**
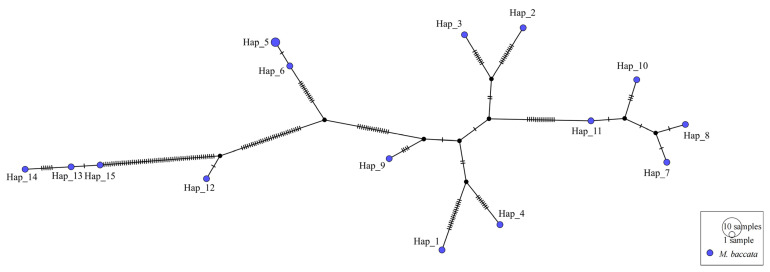
Haplotype analysis of *M. baccata* and its variants. Hap_12, 13, 14 and 15 represent *M. baccata* var. xiaojinensis.

The dominant and shared haplotypes were obtained by haplotype analysis in order to understand the species gene exchange and evolution process. By comparing the different individuals of *M. baccata* and *M. baccata* var. xiaojinensis, it was found that they formed 15 haplotypes and that haplotype 5 was composed of two individuals (*M. baccata* O5 and O6). It can be clearly seen from Figure 9 that the four haplotypes (Hap11, 10, 8 and 7) were closely diffused and evolved and that variants Hap_12, 13, 14 and 15 (*M. baccata* var. xiaojinensis) were obviously far away from the *M. baccata* on the right (Figure 9), which is in accordance with their morphological and evolutionary characteristics.

#### 3.5.3. Phylogenetic Tree

**Figure 10:**
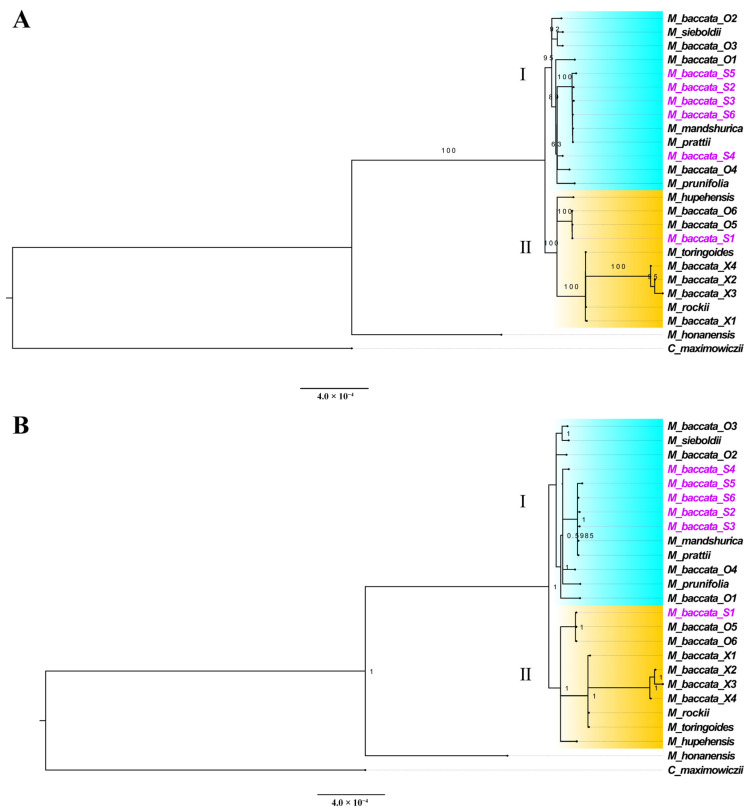
Phylogenetic relationships and species classification of *M. baccata* and *Malus* based on chloroplast genome sequences (126,832 loci). The scale of the phylogenetic tree reflects the evolutionary distance. The genomes and sequences produced in this study are distinguished in purple. I and II represent two branches containing *M. baccata* individuals, highlighted in blue and orange respectively. (**A**) Maximum likelihood tree—the number on the branch indicates the bootstrap support; (**B**) Bayesian inference tree—the number at the node represents the posterior probability.

Based on the whole chloroplast genome sequences (24 *Malus* and 1 *Crataegus* sample), the evolutionary relationships of *Malus* species were compared (*C. maximowiczii* was used as the outgroup to specify the tree root). As shown in Figure 10A, *Malus* forms a large clade in which *M. honanensis* was juxtaposed with other individuals, indicating that it was evolutionarily distant and relatively independent. *M. baccata* O5, O6 and S1, *M. hupehensis*, *M. rockii*, *M. toringoides* and *M. baccata* var. xiaojinensis X1, X2, X3 and X4 together form a branch (II); the remaining individuals evolved into another group (I). The evolutionary network (I and II) could still reflect the diversity of *M. baccata*. The branch of the BI (Bayesian inference) tree is similar to the ML tree, and the posterior probability of each node is high (Figure 10B), indicating that the phylogenetic relationship can be trusted. In addition to the whole genome, the evolution of two single-copy genes (*matK* and *rbcL*) is also discussed (Figure S4). Due to their moderate variation and practical detection, they played an important role in comparing interspecific relationships and species identification. NJ tree and ML tree building with *matK* coding sequences (Figure S4A,C) also described similar topological relationships to the above. Additionally, four *M. baccata* var. xiaojinensis X1~X4, *M. rockii* and *M. toringoides* gathered together (Figure S4A,C), which fully verifies the high unity of their origin distribution and geographical location (southwest region of China).

**Figure 11:**
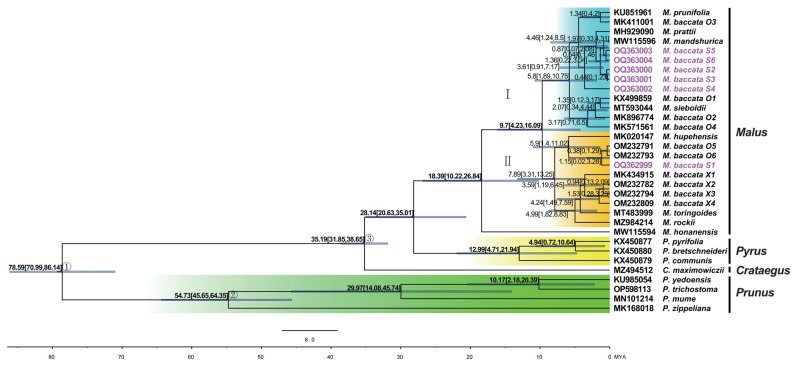
Divergent time tree of *M. baccata* and other species based on shared coding sequences in cp genomes (58,056 loci). The six *M. baccata* individuals in this study are shown in purple. The two evolutionary branches of the *Malus* genus that contain the *M. baccata* (I and II) and the speciation branches of the *Pyrus* and *Prunus* genera are highlighted in blue, orange, yellow and green, respectively. The fossil points and calibration dates are plotted with circled numbers. The scale shows millions of years ago. The numbers and intervals on the nodes represent the divergence time and the 95% highest posterior density (also drawn in blue node bars).

Based on Bayesian inference analysis and the divergence time estimation of chloroplast genes shared by different species (Table S3), it was found that the topology of the evolutionary tree (Figure 11) was consistent with that of the chloroplast genome sequence, and the distribution of *M. baccata* in the genus *Malus* was in two evolutionary clades (I and II). Clade I and II diverged 9.7 million years ago (95% HPD: 4.23~16.09 MYA) and then the two clades continued to evolve. Clade I consists of nine *M. baccata* individuals (S2, S3, S4, S5, S6, O1, O2, O3 and O4); the three *M. baccata* members of II are S1, O5 and O6. In addition, it can be seen from Figure 11 that the divergence time of the three *Pyrus* species was 12.99 MYA (95% HPD: 4.71~21.94 MYA) and the four species (*P. yedoensis*, *P. zippeliana*, *P. mume* and *P. trichostoma*) of *Prunus* diverge at 54.73 MYA (45.65~64.35 MYA, 95% HPD) in evolution.

## 4. Discussion

Chloroplasts exist in the cytoplasm and are relatively independent and semi-autonomous with maternally inherited elements (cp genome) [41]. The chloroplast genome has a double-chained structure, and its size and composition are stable [42]. Due to the influence of gene flow and DNA mutation, the chloroplast genome carries good marker information that can be used as a powerful tool for the study of population evolution, species classification and genetic engineering [43].

As a very important genus in the Rosaceae family, *Malus* includes apple, rootstock and a large number of ornamental crabapple species that have rich germplasm resources and biological value [5]. However, due to extensive hybridization and complex morphology, species classification and germplasm identification of *Malus* are extremely difficult [11]. Since the development of DNA sequencing technology, chloroplast maps of *Malus* had been published successfully, including *M. prattii* [44], *M. sieboldii* [45], *M. toringoides* [46,47], etc. The identification and comparison of these sequences have promoted the systematic classification of *Malus* [48]. In this study, through the assembly, annotation, comparison and phylogeny of chloroplast genomes, we improved the similarity and diversity analysis of the plastid sequences of *M. baccata* and other *Malus* species and systematically described their phylogeographic structures and evolutionary relationships.

The full length of *M. baccata* cp genomes was between 160,083 (S1) and 160,295 (S4), which was similar to previously released versions (*M. baccata* O1~O6). Through sequence homology analysis, it can be seen that *M. baccata* has high similarity to other species of *Malus* (such as *M. hupehensis*, *M. sieboldii* and *M. prunifolia*), and no structural rearrangement was found in these genomes, indicating that the variation is relatively moderate. Through repeat sequence recognition, 93 to 96 SSRs were found in the *M. baccata* cp genome, of which the single nucleotide repeat type accounted for the largest proportion. In addition, dispersed repeats are abundant (57~75), different in different chloroplast genomes and play a role in the stability of cp genomes [49]. A total of 110~112 genes were identified in the *M. baccata* chloroplast based on homologous annotation, including 77~78 CDSs, 29~30 tRNAs and 4 rRNAs. Codon usage pattern analysis revealed that the chloroplast genes of *M. baccata* had a certain degree of bias, for example, GCU appeared more frequently in Ala (alanine) and the stop codon (TER) preferred to use UAA rather than UAG and UGA. Similar results have been supported in previous studies; they found that the RSCU values of UUA, GCU and AGA are the most preferred ones [50]. As components of the chloroplast genome, IR regions were more stable than SC regions [51]. This phenomenon is fully demonstrated both in the characterization of GC content and in the calculation of nucleic acid polymorphism and mutation hotspots at the whole-genome level. There were several hypervariable regions in the chloroplast genome of *M. baccata* and other *Malus* species (*trnR-UCU*-*atpA*, *trnT/UGU*-*trnL/UAA*, *rps16* and *nhdD*), which provide the genetic basis for the development of interspecific molecular markers.

*M. baccata* is a wild species of *Malus* that is native to China. It is widely distributed in the north and southwest of China with abundant variation types and has important ecological and breeding significance [3,4,6,7]. Based on the variations of different *M. baccata* individuals, the phylogeographic relationship was mainly divided into three categories: (1) S1, O6 and O5; (2) O3, O2, O4, S4 and O1; and (3) S2, S5, S3 and S6. The phylogenetic tree and principal component analysis all support the result of structure differentiation. The geographical and systematic distribution of the species can be well explained by the analysis of haplotype variation. A past study found that two haplotypes of the apple were shared by several wild species [13]. By further comparing the evolution of *M. baccata* and its variant (*M. baccata* var. xiaojinensis), it was found that *M. baccata* var. xiaojinensis was far away from the main haplotype of *M. baccata* and there were differences in the clade. Previous studies have shown that *M. baccata* var. xiaojinensis is closely related to *M. hupehensis* in comparisons of 13 chloroplast genomes [52]. In our data, *M. baccata* var. xiaojinensis and *M. toringoides* in the evolutionary branch cluster together, indicating that they are similar in maternal inheritance. Due to the complexity of morphological characteristics and evolutionary history, more data are needed to explain the classification and attribution of *M. baccata* var. xiaojinensis.

In addition, the differentiation of *M. baccata* was characterized by maximum likelihood and Bayesian inference. The results showed that from the comparison of the whole cp genome and the shared cp genes, different members of *M. baccata* formed two large branches (I and II). In the phylogenetic and clustering analysis (SSR markers) of 798 *Malus* resources native to China, it was found that the population of *M. baccata* is dispersed in different evolutionary branches [6]. The date of divergence between the two main branches of *M. baccata* and *M. honanensis* dates back 18.39 MYA (95% HPD: 10.22~26.84 MYA), which is close to that previously reported using 47 chloroplast genomes (16.66~30.29 MYA) [15].

The assembly and comparative analysis of the chloroplast genomes of *M. baccata* are necessary to understand its genetic variation and phylogeographic relationship. Therefore, the results of this study can provide valuable references for species identification and breeding engineering of the *Malus* genus, so as to facilitate the work of germplasm resources and conservation biology.

## 5. Conclusions

Six complete chloroplast genomes of *Malus baccata* were obtained by whole-genome resequencing and sequence assembly, with whole lengths ranging from 160,083 to 160,295 bp. Compared with other *Malus* species, it was found that there were more sites of polymorphisms and hotspots of variation in LSC and SSC regions; however, IR regions were stable due to high GC content and sequence similarity. Through repeat sequence and highly variable region recognition, numerous mononucleotide SSRs were found in the *M. baccata* cp genome, and *trnG-UCC*-*trnR-UCU*, *trnR-UCU*-*atpA*, *trnT-UGU*-*trnL-UAA*, *ndhC*-*trnV-UAC*, *ndhF*-*rpl32* and *ccsA*-*ndhD* could be selected as candidate markers for distinguishing between different species of *Malus*. According to the analysis of phylogeny and haplotype, *M. baccata* formed two main branches (I and II) after the differentiation of *Malus* genus, and the variant (*M. baccata* var. xiaojinensis) was different from that of *M. baccata* to some extent. The results of the diverging time estimates suggest that clade I and II diverged 9.7 MYA. The chloroplast genome and its variation data for *M. baccata* provided a good resource for the species classification of *Malus*.

## Figures and Tables

**Table 1:** Comparison of the characteristics of 12 chloroplast genomes from *M. baccata*.

Code	GenBank ID	Length (bp)				GC Content (%)			
		Total	LSC	SSC	IRs	Total	LSC	SSC	IRs
S1	OQ362999	160,083	88,190	19,187	26,353	36.5	34.2	30.4	42.7
S2	OQ363000	160,207	88,323	19,176	26,354	36.5	34.2	30.4	42.7
S3	OQ363001	160,189	88,305	19,176	26,354	36.5	34.2	30.4	42.7
S4	OQ363002	160,295	88,409	19,178	26,354	36.5	34.2	30.4	42.7
S5	OQ363003	160,197	88,314	19,175	26,354	36.5	34.2	30.4	42.7
S6	OQ363004	160,199	88,315	19,176	26,354	36.5	34.2	30.4	42.7
O1 *	KX499859.1	160,163	88,267	19,188	26,354	36.5	34.2	30.4	42.7
O2 *	MK896774.1	160,024	88,134	19,182	26,354	36.6	34.2	30.4	42.7
O3 *	MK411001.1	160,088	88,198	19,182	26,354	36.6	34.2	30.4	42.7
O4 *	MK571561.1	160,149	88,260	19,181	26,354	36.5	34.2	30.4	42.7
O5 *	OM232791.1	160,029	88,143	19,180	26,353	36.6	34.2	30.4	42.7
O6 *	OM232793.1	160,116	88,223	19,187	26,353	36.6	34.2	30.4	42.7

* Represents previously reported *M. baccata* species on the NCBI website.

**Table 2:** Comparison of annotations on the chloroplast genomes of *M. baccata*.

Sample	SSR (Repeat Sequence)				Including Repetition (Gene)				Eliminate Redundancy (Gene)			
	Mono-	Di-	Tetra-	Penta-	Total	CDS	tRNA	rRNA	Total	CDS	tRNA	rRNA
S1	70	18	7	1	127	84	35	8	111	78	29	4
S2	68	19	7	0	128	83	37	8	111	77	30	4
S3	67	19	7	0	127	84	35	8	111	78	29	4
S4	69	19	7	0	129	84	37	8	112	78	30	4
S5	67	19	7	0	128	83	37	8	111	77	30	4
S6	68	19	7	0	126	83	35	8	110	77	29	4

**Table 3:** Codon usage characteristics of cp genes in *M. baccata*.

Sample	T3s	C3s	A3s	G3s	CAI	CBI	FOP	ENC	GC3s	GC
S1	0.4686	0.1689	0.4328	0.1806	0.166	−0.107	0.35	49.53	0.267	0.378
S2	0.4688	0.1688	0.433	0.1805	0.165	−0.106	0.351	49.51	0.267	0.378
S3	0.469	0.1689	0.4326	0.1806	0.166	−0.106	0.351	49.51	0.267	0.378
S4	0.4688	0.1689	0.4327	0.1807	0.166	−0.106	0.35	49.52	0.267	0.378
S5	0.4689	0.1689	0.4327	0.1807	0.166	−0.106	0.351	49.52	0.267	0.378
S6	0.4688	0.1689	0.4328	0.1806	0.166	−0.106	0.351	49.51	0.267	0.378

T3s, C3s, A3s and G3s represent the base (T, C, A and G, respectively) content at the synonymous third codon position. CAI (codon adaptation index), CBI (codon bias index), FOP (frequency of optimal codons), ENC (effective number of codons), GC3s (GC of silent 3rd codon posit), GC (GC content of gene).

## Data Availability

The data presented in the study are deposited in the NCBI repository, accession numbers: OQ362999, OQ363000, OQ363001, OQ363002, OQ363003 and OQ363004.

## Supplementary Materials

The following supporting information can be downloaded at: https://www.mdpi.com/article/10.3390/biom13060962/s1, Figure S1: GC content and skew in the chloroplast genomes of *M. baccata*; Figure S2: Similar sequences of cp genomes in *M. baccata* and other *Malus* species; Figure S3: Hotspots of variation in the chloroplast genome of *Malus;* Figure S4: Evolutionary analyses of *M. baccata* and different *Malus* species based on single-copy genes (*matK* and *rbcL*); Table S1: The sample information for the *M. baccata* germplasms used in this study and the assembly quality of the chloroplast genomes; Table S2: Names of chloroplast coding genes with introns and their genome distribution in *M. baccata*; Table S3: Chloroplast gene information shared by different species for Bayesian inference analysis and divergence time estimation.

## Abbreviations

IR	inverted repeat region
LSC	large single-copy region
SSC	small single-copy region
IRA	inverted repeat region A
IRB	inverted repeat region B
CDS	coding sequence
tRNA	transfer RNA
rRNA	ribosomal RNA
SNP	single nucleotide polymorphism
INDEL	insertion and deletion
GTR	general time reversible
ESS	effective sample size
MCMC	Markov chain Monte Carlo
MYA	million years ago
Cp	chloroplast
SSR	simple sequence repeats
LTR	long tandem repeats
LSR	large sequence repeats
Pi	nucleotide diversity
JLB	junction site of LSC–IRb
JSB	junction site of IRb–SSC
JSA	junction site of SSC–IRa
JLA	junction site of IRa–LSC
PCA	principal component analysis
NJ	neighbor joining
ML	maximum likelihood
BI	Bayesian inference
HPD	highest posterior density
