# Supplementary material for: Molecular Structure and Variation Characteristics of the Plastomes from Six Malus baccata (L.) Borkh. Individuals and Comparative Genomic Analysis with Other Malus Species
Source: Biomolecules. 2023 Jun 8;13(6):962. doi: 10.3390/biom13060962 (PMC10296354; doi:10.3390/biom13060962)
Supplement: Supplementary file 1 [file biomolecules-13-00962-s001.zip › biomolecules-2376563-supplementary.pdf]

biomolecules

MDPI

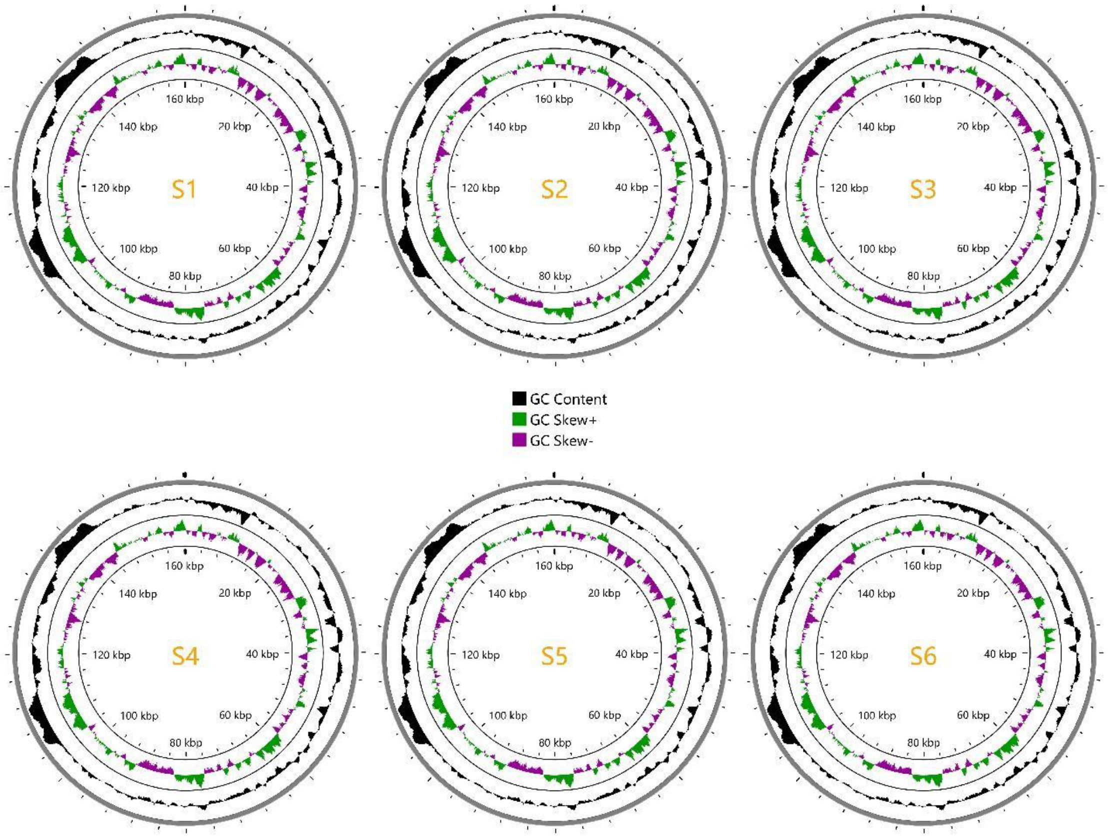

Figure S1. GC content and skew in the chloroplast genomes of *M. baccata*.

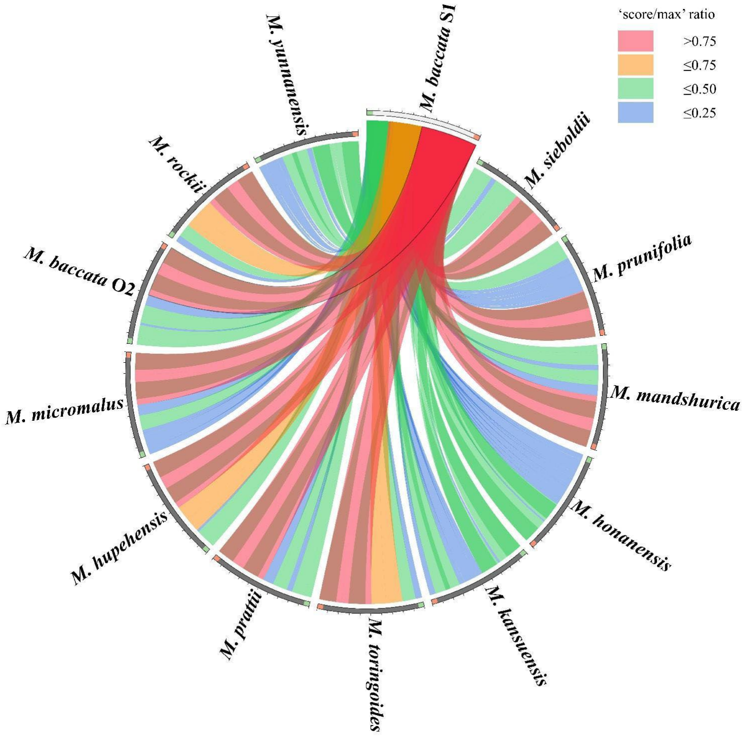

Figure S2. Similar sequences of cp genomes in *M. baccata* and other *Malus* species. Different colors are added to the alignment position of different scores, as shown in the legend.

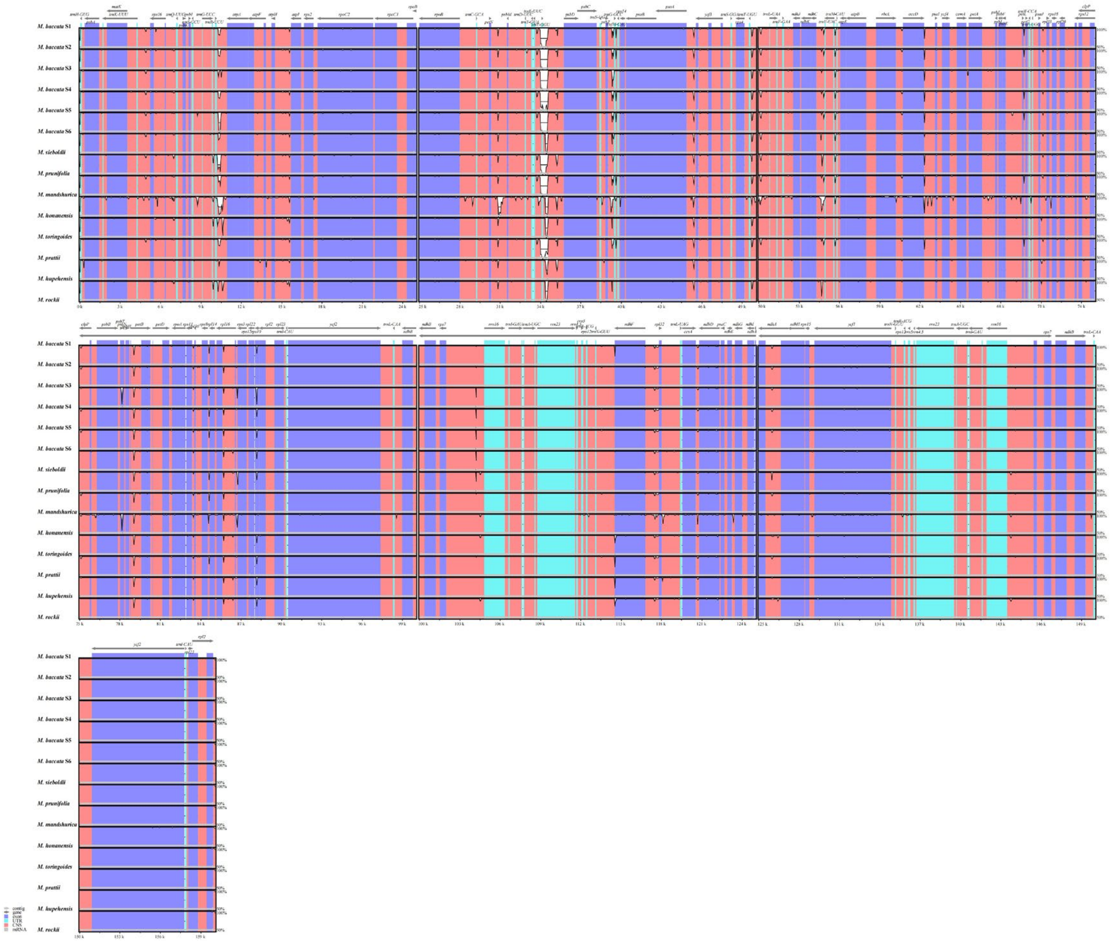

Figure S3. Hotspots of variation in the chloroplast genome of *Malus*. The vertical axis represents the cp genome identity within 50%~100%. Blue area indicates the location of the gene, and pink blocks indicate conserved non-coding sequences.

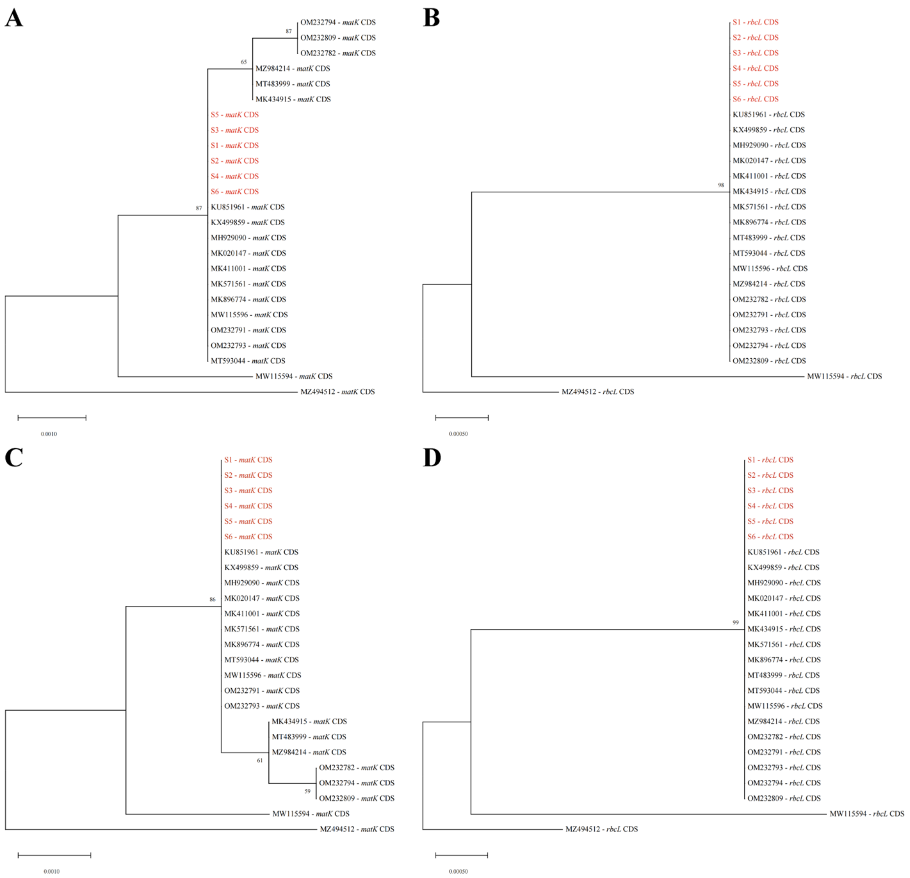

Figure S4. Evolutionary analyses of *M. baccata* and different *Malus* species based on single-copy genes (*matK* and *rbcL*). (**A,B**) Neighbor-joining method; (**C,D**) Maximum-likelihood method. The sequences submitted in this study were marked in red.

Table S1. The sample information for the *M. baccata* germplasms used in this study and the assembly quality of the chloroplast genomes.

Sample Code	Collection Sites	Geographical Origin	Total Reads	Assembled Reads	Average Insert Size	Average kmer-coverage	Average Base-coverage
S1 (*M. baccata*)	Qingdao (36°24′N, 120°54′E)	Shanxi province, China (37°78′N, 112°58′E)	15,157,854	477,929	357.218	120.7	393.6
S2 (*M. baccata*)	Qingdao (36°24′N, 120°54′E)	Nei monggol autonomous region, China (40°77′N, 111°67′E)	27,185,718	708,622	410.414	131.7	429.3
S3 (*M. baccata*)	Qingdao (36°24′N, 120°54′E)	Shandong province, China (36°71′N, 117°08′E)	27,154,474	500,977	404.132	129.2	421.3
S4 (*M. baccata*)	Qingdao (36°24′N, 120°54′E)	Shandong province, China (36°71′N, 117°08′E)	16,098,453	417,557	305.23	114.3	373.7
S5 (*M. baccata*)	Qingdao (36°24′N, 120°54′E)	Hebei province, China (38°03′N, 114°56′E)	19,051,684	410,700	314.258	112.4	367.3
S6 (*M. baccata*)	Qingdao (36°24′N, 120°54′E)	Heilongjiang province, China (45°69′N, 126°62′E)	20,317,230	421,875	339.891	114.1	372.1

Table S2. Names of chloroplast coding genes with introns and their genome distribution.

Gene Name	Location	Genome Distribution						Intron Number
		S1	S2	S3	S4	S5	S6	
rps16	LSC	5243..6367	5258..6388	5258..6381	5255..6379	5259..6382	5258..6382	1
atpF	LSC	12526..13813	12556..13841	12548..13833	12543..13829	12549..13833	12541..13826	1
rpoC1	LSC	21958..24744	21977..24763	21968..24754	21965..24772	21968..24775	21961..24747	1
ycf3	LSC	45588..47546	45687..47646	45670..47629	45747..47706	45678..47637	45671..47630	2
clpP	LSC	73799..75842	73931..75968	73914..75950	73980..76022	73922..75959	73923..75960	2
petB	LSC	78780..80247	78906..80350	78888..80332	78960..80404	78897..80341	78898..80342	1
petD	LSC	80438..81644	80541..81747	80523..81729	80595..81801	80532..81738	80533..81739	1
rpl16	LSC	85194..86576	85313..86708	85295..86690	85366..86763	85304..86699	85306..86701	1
rpl2	IRB	88381..89891	88514..90024	88496..90006	88600..90110	88505..90015	88506..90016	1
ndhB	IRB	98993..101194	99126..101273	99108..101309	99212..101413	99117..101318	99118..101319	1
ndhA	SSC	124965..127189	125092..127311	125074..127293	125180..127400	125083..127301	125084..127303	1
ndhB	IRA	147080..149281	147258..149405	147186..149387	147292..149493	147194..149395	147196..149397	1
rpl2	IRA	158383..159893	158507..160017	158489..159999	158595..160105	158497..160007	158499..160009	1
rps12	LSC-IRB/LSC-IRA	73521..102837/ 73521..146238	73653..102970/ 73653..146362	73636..102949/ 73636..146344	73702..103056/ 73702..146450	73644..102961/ 73644..146352	73645..102962/ 73645..146354	2

Table S3. Chloroplast gene information shared by different species for Bayesian inference analysis and divergence time estimation.

Gene Group	Gene Code	Gene Name
Subunit of Acetyl-CoA-carboxylase	*acc*	*accD*
Subunits of ATP synthase	*atp*	*atpA, atpB, atpE, atpF, atpH, atpI*
c-type cytochrome synthesis gene	*ccs*	*ccsA*
Envelop membrane protein	*cem*	*cemA*
Protease	*clp*	*clpP*
Maturase	*mat*	*matK*
Subunits of NADH-dehydrogenase	*ndh*	*ndhA, ndhB, ndhC, ndhD, ndhE, ndhF, ndhG, ndhH, ndhI, ndhJ, ndhK*
Subunits of cytochrome b/f complex	*pet*	*petA, petB, petD, petG, petL, petN*
Subunits of photosystem I	*psa*	*psaA, psaB, psaJ*
Subunits of photosystem II	*psb*	*psbA, psbB, psbC, psbD, psbE, psbF, psbH, psbJ, psbK, psbM, psbT, psbZ*
Subunit of RuBisCO	*rbc*	*rbcL*
Large subunits of ribosome	*rpl*	*rpl2, rpl14, rpl16, rpl20, rpl22, rpl23, rpl32, rpl33, rpl36*
DNA-dependent RNA polymerase	*rpo*	*rpoA, rpoB, rpoC1, rpoC2*
Small subunits of ribosome	*rps*	*rps3, rps4, rps7, rps8, rps11, rps14, rps15, rps16, rps19*
Conserved open reading frames	*ycf*	*ycf1**

*For *ycf1* gene, only the longest one (non-pseudogene) was reserved and selected for phylogenetic tree analysis.
